# Crystal structure and Hirshfeld surface analysis of (5a*S*,8a*R*)-3,5a-dimethyl-8-methyl­idene-2-oxododeca­hydro­oxireno[2′,3′:6,7]naphtho­[1,2-*b*]furan-6-yl (*Z*)-2-methyl­but-2-enoate extracted from *Ferula persica*

**DOI:** 10.1107/S205698902300333X

**Published:** 2023-04-21

**Authors:** Elvin G. Karimli, Victor N. Khrustalev, Margarita N. Kurasova, Mehmet Akkurt, Ali N. Khalilov, Ajaya Bhattarai, İbrahim G. Mamedov

**Affiliations:** a Azerbaijan Medical University, Bakikhanov st. 23, AZ1022, Baku, Azerbaijan; b Peoples’ Friendship University of Russia (RUDN University), Miklukho-Maklay St. 6, Moscow, 117198, Russian Federation; c N. D. Zelinsky Institute of Organic Chemistry RAS, Leninsky Prosp. 47, Moscow, 119991, Russian Federation; d Department of Physics, Faculty of Sciences, Erciyes University, 38039 Kayseri, Türkiye; e "Composite Materials" Scientific Research Center, Azerbaijan State Economic University (UNEC), H. Aliyev str. 135, Az 1063, Baku, Azerbaijan; f Department of Chemistry, Baku State University, Z. Khalilov str. 23, Az, 1148, Baku, Azerbaijan; g Department of Chemistry, M.M.A.M.C (Tribhuvan University) Biratnagar, Nepal; Vienna University of Technology, Austria

**Keywords:** crystal structure, hydrogen bonds, sesquiterpene lactones, *Ferula persica*, Hirshfeld surface analysis

## Abstract

In the title compound, C_20_H_26_O_5_, the two cyclo­hexane rings adopt boat and half-chair conformations. In the crystal, adjacent mol­ecules are connected by inter­molecular C—H⋯O hydrogen bonds, forming a three-dimensional network. According to a Hirshfeld surface study, H⋯H inter­actions are the most significant contributors to the crystal packing (63.0%).

## Abstract 2

In the crystal of the title compound, adjacent mol­ecules are connected by inter­molecular C—H⋯O hydrogen bonds, forming a three-dimensional network.

## 1. Chemical context

Sesquiterpene lactones are a significant group of natural products isolated from the extracts of various parts of medicinal plants. As a medicinal plant, the *Ferula* genus is rich in coumarins, specifically sesquiterpene coumarins. *Ferula* species are found in the Mediterranean region, Central Asia, Siberia, China, Afghanistan, Iran, North Africa and the Caucasus (Mir-Babayev & Houghton, 2002 ▸). The members of this genus typically have a heavy fragrance due to the presence of essential oils or oleoresins in their content. This genus is applied for the cure of various organ disorders in folk medicine (Salehi *et al.*, 2019 ▸). These herbs have been used for oleo-gum resin, plant extracts, and essential oils. Moreover, the essential oils and extracts of different species of this herb can be used as natural food preservatives due to their anti­oxidant and anti­microbial activity (Daneshniya *et al.*, 2021 ▸). 
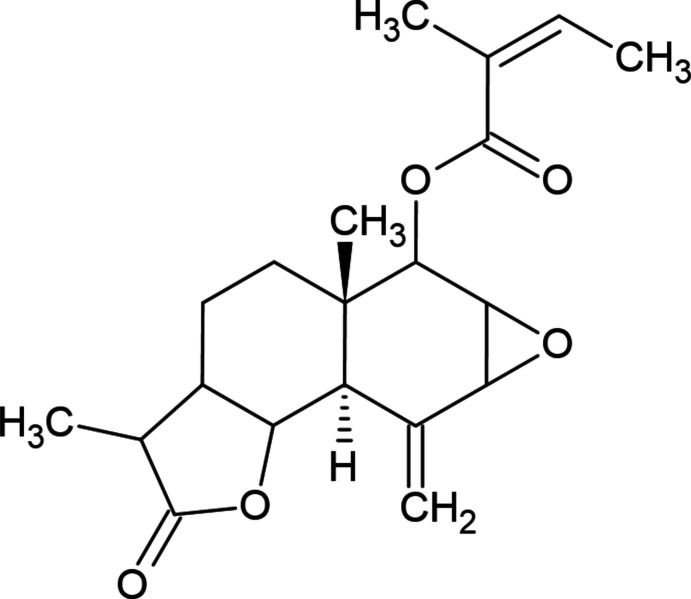


Herein, in the framework of our ongoing structural studies, we report the crystal structure and Hirshfeld surface analysis of the title compound, (5a*S*,8a*R*)-3,5a-dimethyl-8-methylid­ene-2-oxododeca­hydro­oxireno[2′,3′:6,7]naphtho­[1,2-*b*]furan-6-yl (*Z*)-2-methyl­but-2-enoate extracted from *Ferula persica*.

## 2. Structural commentary

**Figure 1:**
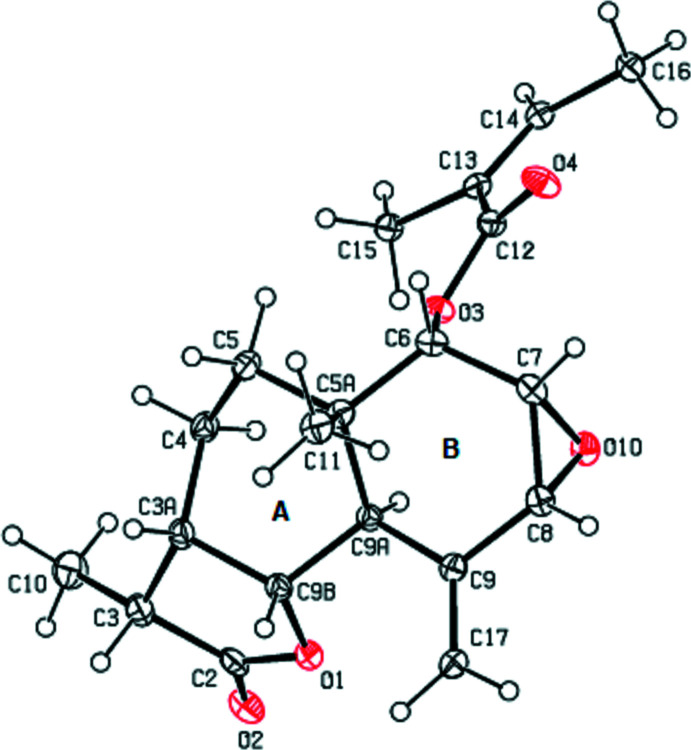
The mol­ecular structure of the title compound, showing the atom labelling and displacement ellipsoids drawn at the 30% probability level.

A view of the mol­ecular structure of the title compound is shown in Fig. 1 ▸. The cyclo­hexane rings (*A*: C3*A*/C4/C5/C5*A*/C9*A*/C9*B*; *B*: C5*A*/C6–C9/C9*A*) adopt boat and half-chair conformations, respectively. The puckering parameters (Cremer & Pople, 1975 ▸) of the *A* and *B* rings are *Q*_T_ = 0.7259 (19) Å, θ = 83.29 (15)°, φ = 51.45 (15)°, and *Q*_T_ = 0.5337 (18) Å, θ = 52.1 (2)°, φ = 331.7 (3)°, respectively.

## 3. Supra­molecular features and Hirshfeld surface analysis

**Figure 2:**
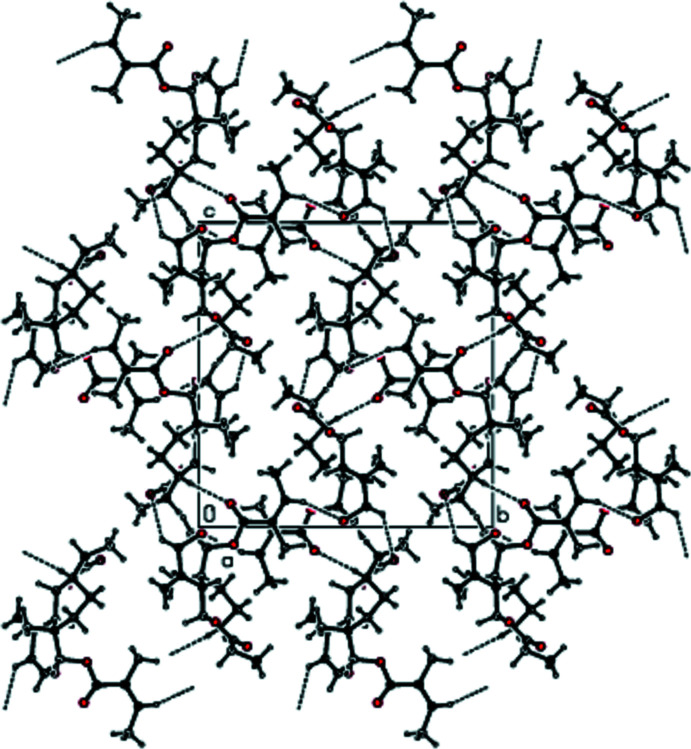
View of the packing of the title compound down the *a* axis.

In the crystal of the title compound, adjacent mol­ecules are connected by inter­molecular C—H⋯O hydrogen bonds, forming a three dimensional network (Tables 1 ▸ and 2 ▸). Figs. 2 ▸, 3 ▸ and 4 ▸ show packing views of the title compound down the *a*, *b* and *c* axes, respectively.

**Figure 5:**
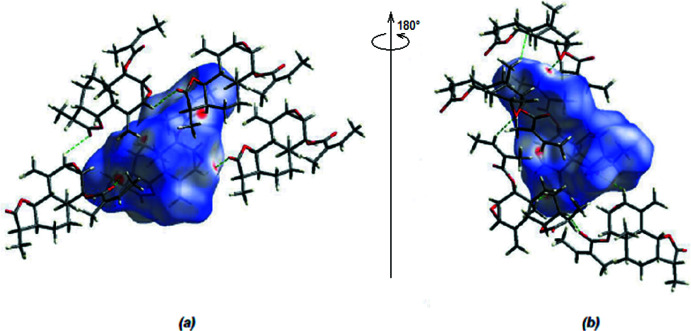
(*a*) Front and (*b*) back sides of the three-dimensional Hirshfeld surface of the title compound mapped over *d*_norm_, with a fixed colour scale of −0.1633 to +1.3364 a.u.

*CrystalExplorer17* (Spackman *et al.*, 2021 ▸) was used to compute Hirshfeld surfaces of the title mol­ecule. The *d*_norm_ mappings for the mol­ecule were performed in the range −0.1633 to +1.3364 a.u. The locations of the C—H⋯O inter­actions are shown by intense red circles on the *d*_norm_ surface (Fig. 5 ▸ *a*,*b*).

**Figure 6:**
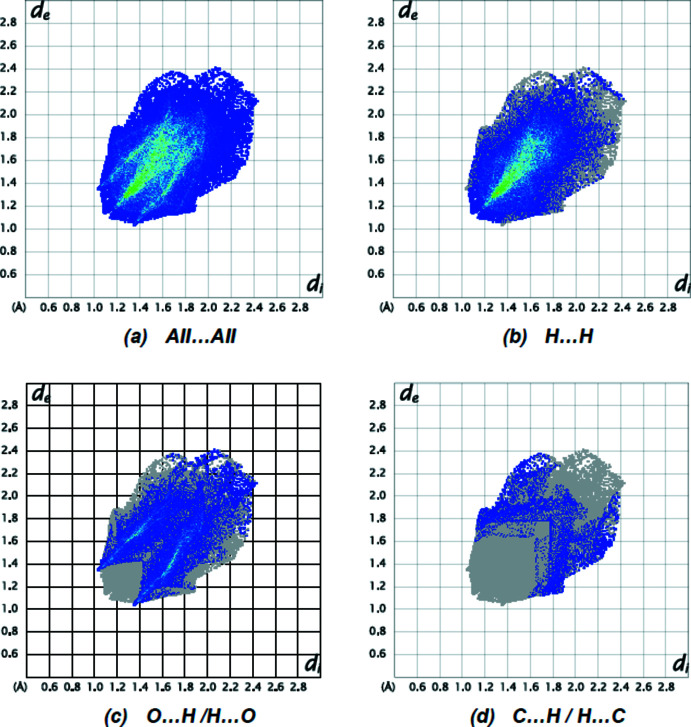
The two-dimensional fingerprint plots of the title compound, showing (*a*) all inter­actions, and delineated into (*b*) H⋯H, (*c*) O⋯H/H⋯O and (*d*) C⋯H/H⋯C inter­actions. [*d*_e_ and *d*_i_ represent the distances from a point on the Hirshfeld surface to the nearest atoms outside (external) and inside (inter­nal) the surface, respectively].

Fig. 6 ▸ shows the full two-dimensional fingerprint plots for the mol­ecule and those delineated into the major contacts. H⋯H inter­actions (Fig. 6 ▸ *b*; 63.0% contribution) are the major contributor to the crystal packing with O⋯H/H⋯O (Fig. 6 ▸ *c*; 28.3%) and C⋯H/H⋯C (Fig. 6 ▸ *d*; 7.5%) inter­actions representing the next highest contributions. The percentage contributions of comparatively weaker inter­actions are O⋯C/C⋯O (0.5%) , O⋯O (0.4%) and C⋯C (0.3%). Relevant short inter­molecular atomic contacts are summarized in Table 2 ▸.

## 4. Database survey

Two closely related compounds are 1β-angelo­yloxy-2β,3β-ep­oxy-5β*H*,7α*H*-l0α-methyl­eudesma-4(15),11(13)-dien-6,12-olide (**I**) (Rychlewska *et al.*, 1992 ▸) and 1β-angelo­yloxy-5β*H*,6α*H*,7α*H*,11α*H*-10α-methyl­eudesma-2,4(15)-dien-6,12-olide (**II**) (Rychlewska *et al.*, 1992 ▸).

The largest difference between the two structures (**I** and **II**) lies in the cyclo­hexane *B* ring, which is of the rigid-chair type in **I** and of the flexible boat type in **II**. In both crystal structures, the mol­ecules are held together mostly by van der Waals forces.

## 5. Synthesis and crystallization

The title compound has previously been isolated from the roots of the *Ferula oopoda* plant and fully characterized (Serkerov, 1972 ▸). The compound used for the current study was isolated from the roots of the *Ferula persica* herb by a similar method.

## 6. Refinement

Crystal data, data collection and structure refinement details are summarized in Table 3 ▸. H atoms of the –C=CH_2_ group were located in a difference-Fourier map and refined freely [C17—H17*A* = 0.94 (2) Å, C17—H17*B* = 0.97 (2) Å]. All other H atoms were placed at calculated positions and refined using a riding model, with C—H = 0.95–1.00 Å, and with *U*_iso_(H) = 1.2 or 1.5*U*_eq_(C). The remaining maximum electron density peak (0.56 e^−^ Å^−3^) is 1.41 Å away from C17 and the minimum density peak (–0.16 e Å^−3^) is 0.92 Å away from C9.

## Supplementary Material

Crystal structure: contains datablock(s) I, global. DOI: 10.1107/S205698902300333X/wm5679sup1.cif

Structure factors: contains datablock(s) I. DOI: 10.1107/S205698902300333X/wm5679Isup2.hkl

CCDC reference: 2255517

Additional supporting information:  crystallographic information; 3D view; checkCIF report

## Figures and Tables

**Figure 3:**
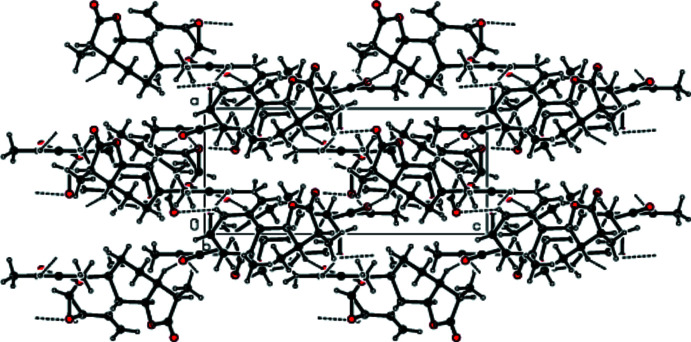
View of the packing of the title compound down the *b* axis.

**Figure 4:**
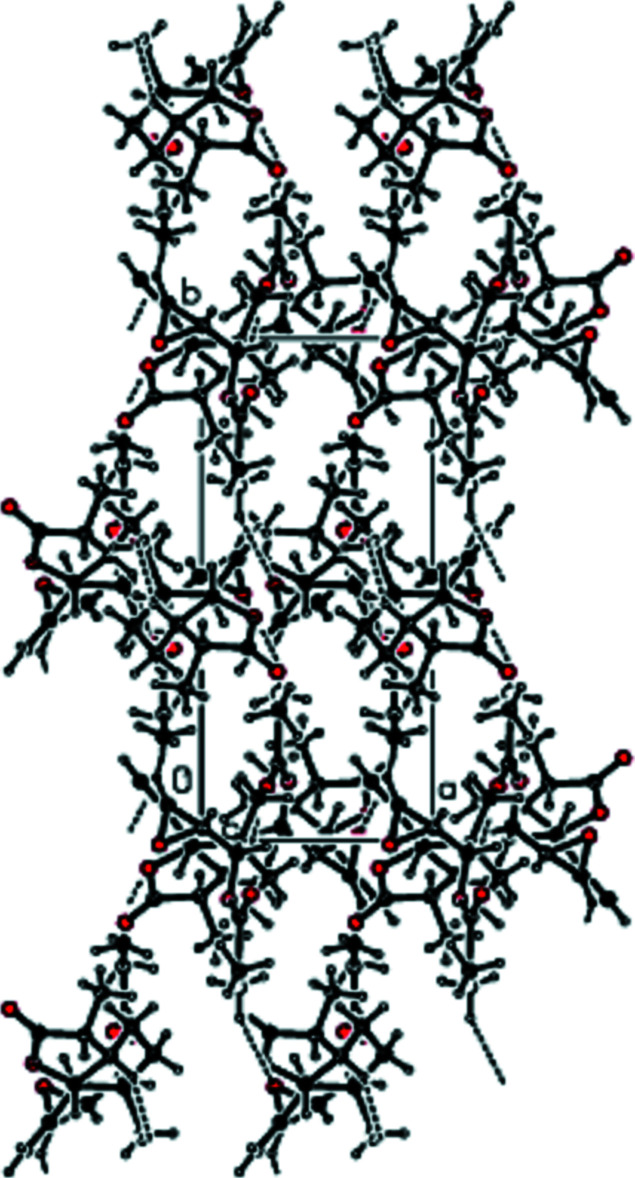
View of the packing of the title compound down the *c* axis.

**Table 1:** Hydrogen-bond geometry (Å, °)

*D*—H⋯*A*	*D*—H	H⋯*A*	*D*⋯*A*	*D*—H⋯*A*
C3*A*—H3*A*⋯O4^i^	1.00	2.45	3.353 (2)	149
C8—H8⋯O2^ii^	1.00	2.50	3.176 (2)	124
C14—H14⋯O10^iii^	0.95	2.57	3.423 (2)	149

Symmetry codes: (i) 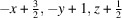 ; (ii) 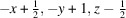 ; (iii) 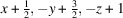 .

**Table 2:** Summary of short inter­atomic contacts (Å) in the title compound

H15*A*⋯H16*C*	2.39	− 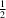 + *x*, 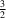 − *y*, 1 − *z*
O2⋯H8	2.50	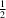 − *x*, 1 − *y*, 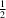 + *z*
O4⋯H3*A*	2.45	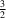 − *x*, 1 − *y*, − 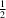 + *z*
H8⋯H10*A*	2.48	1 − *x*, − 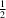 + *y*, 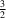 − *z*
H11*B*⋯H17*A*	2.40	1 + *x*, *y*, *z*

**Table 3:** Experimental details

Crystal data	
Chemical formula	C_20_H_26_O_5_
*M*_r_	346.41
Crystal system, space group	Orthorhombic, *P*2_1_2_1_2_1_
Temperature (K)	100
*a*, *b*, *c* (Å)	7.11296 (5), 15.4597 (10), 16.0358 (10)
*V* (Å^3^)	1763.36 (16)
*Z*	4
Radiation type	Cu *K*α
μ (mm^−1^)	0.76
Crystal size (mm)	0.21 × 0.18 × 0.13

Data collection	
Diffractometer	XtaLAB Synergy, Dualflex, HyPix
Absorption correction	Gaussian (*CrysAlis PRO*; Rigaku OD, 2022 ▸)
*T*_min_, *T*_max_	0.674, 1.000
No. of measured, independent and observed [*I* > 2σ(*I*)] reflections	19755, 3751, 3717
*R*_int_	0.024
(sin θ/λ)_max_ (Å^−1^)	0.634

Refinement	
*R*[*F* ^2^ > 2σ(*F* ^2^)], *wR*(*F* ^2^), *S*	0.031, 0.082, 1.05
No. of reflections	3751
No. of parameters	238
H-atom treatment	H atoms treated by a mixture of independent and constrained refinement
Δρ_max_, Δρ_min_ (e Å^−3^)	0.56, −0.16
Absolute structure	Flack *x* determined using 1576 quotients [(*I* ^+^)−(*I* ^−^)]/[(*I* ^+^)+(*I* ^−^)] (Parsons et al, 2013 ▸).
Absolute structure parameter	0.01 (4)

Computer programs: *CrysAlis PRO* (Rigaku OD, 2022 ▸), SHELXT (Sheldrick, 2015*a* ▸), *SHELXL* (Sheldrick, 2015*b* ▸), *ORTEP-3 for Windows* (Farrugia, 2012 ▸), *PLATON* (Spek, 2020 ▸).

## supplementary crystallographic information

### Crystal data

**Table:**

C_20_H_26_O_5_	*D*_x_ = 1.305 Mg m^−^^3^
*M**_r_* = 346.41	Cu *K*α radiation, λ = 1.54184 Å
Orthorhombic, *P*2_1_2_1_2_1_	Cell parameters from 16716 reflections
*a* = 7.11296 (5) Å	θ = 3.9–77.3°
*b* = 15.4597 (10) Å	µ = 0.76 mm^−^^1^
*c* = 16.0358 (10) Å	*T* = 100 K
*V* = 1763.36 (16) Å^3^	Prism, yellow
*Z* = 4	0.21 × 0.18 × 0.13 mm
*F*(000) = 744	

### Data collection

**Table:**

XtaLAB Synergy, Dualflex, HyPix diffractometer	3717 reflections with *I* > 2σ(*I*)
Radiation source: micro-focus sealed X-ray tube	*R*_int_ = 0.024
φ and ω scans	θ_max_ = 77.8°, θ_min_ = 4.0°
Absorption correction: gaussian (CrysAlis PRO; Rigaku OD, 2022)	*h* = −9→8
*T*_min_ = 0.674, *T*_max_ = 1.000	*k* = −19→16
19755 measured reflections	*l* = −18→20
3751 independent reflections	

### Refinement

**Table:**

Refinement on *F*^2^	Secondary atom site location: difference Fourier map
Least-squares matrix: full	Hydrogen site location: mixed
*R*[*F*^2^ > 2σ(*F*^2^)] = 0.031	H atoms treated by a mixture of independent and constrained refinement
*wR*(*F*^2^) = 0.082	*w* = 1/[σ^2^(*F*_o_^2^) + (0.0444*P*)^2^ + 0.4585*P*] where *P* = (*F*_o_^2^ + 2*F*_c_^2^)/3
*S* = 1.05	(Δ/σ)_max_ < 0.001
3751 reflections	Δρ_max_ = 0.56 e Å^−^^3^
238 parameters	Δρ_min_ = −0.16 e Å^−^^3^
0 restraints	Absolute structure: Flack *x* determined using 1576 quotients [(*I*^+^)-(*I*^-^)]/[(*I*^+^)+(*I*^-^)] (Parsons et al, 2013).
Primary atom site location: difference Fourier map	Absolute structure parameter: 0.01 (4)

### Special details

**Table:**

Geometry. All esds (except the esd in the dihedral angle between two l.s. planes) are estimated using the full covariance matrix. The cell esds are taken into account individually in the estimation of esds in distances, angles and torsion angles; correlations between esds in cell parameters are only used when they are defined by crystal symmetry. An approximate (isotropic) treatment of cell esds is used for estimating esds involving l.s. planes.

### Fractional atomic coordinates and isotropic or equivalent isotropic displacement parameters (Å^2^)

**Table:**

	*x*	*y*	*z*	*U*_iso_*/*U*_eq_	
O1	0.27214 (18)	0.55551 (9)	0.81184 (8)	0.0258 (3)	
O2	0.1708 (2)	0.66397 (9)	0.89164 (9)	0.0325 (3)	
O3	0.62078 (17)	0.61814 (8)	0.55679 (8)	0.0217 (3)	
O4	0.7139 (2)	0.60760 (8)	0.42338 (8)	0.0316 (3)	
C2	0.2969 (3)	0.61638 (12)	0.87107 (10)	0.0257 (4)	
C3	0.4949 (3)	0.61151 (12)	0.90431 (11)	0.0278 (4)	
H3	0.4936	0.5693	0.9516	0.033*	
C3A	0.6045 (3)	0.56914 (11)	0.83285 (11)	0.0237 (4)	
H3A	0.7026	0.5300	0.8568	0.028*	
C4	0.6965 (3)	0.62980 (11)	0.76998 (11)	0.0259 (4)	
H4A	0.7893	0.6675	0.7983	0.031*	
H4B	0.6003	0.6670	0.7434	0.031*	
C5	0.7941 (3)	0.57422 (12)	0.70428 (12)	0.0270 (4)	
H5A	0.8994	0.5429	0.7311	0.032*	
H5B	0.8483	0.6127	0.6612	0.032*	
C5A	0.6636 (2)	0.50725 (11)	0.66081 (11)	0.0220 (3)	
C6	0.6543 (3)	0.52614 (11)	0.56680 (11)	0.0226 (3)	
H6	0.7798	0.5124	0.5420	0.027*	
C7	0.5062 (3)	0.47385 (11)	0.52056 (11)	0.0234 (4)	
H7	0.5477	0.4454	0.4677	0.028*	
C8	0.3552 (3)	0.42987 (11)	0.56746 (11)	0.0227 (3)	
H8	0.3055	0.3750	0.5428	0.027*	
C9	0.3431 (2)	0.43826 (11)	0.65931 (11)	0.0217 (3)	
C9A	0.4598 (2)	0.51073 (10)	0.69607 (10)	0.0194 (3)	
H9A	0.4033	0.5660	0.6756	0.023*	
C9B	0.4515 (2)	0.51404 (11)	0.79115 (10)	0.0219 (3)	
H9B	0.4543	0.4540	0.8143	0.026*	
O10	0.31873 (19)	0.50878 (8)	0.52107 (8)	0.0254 (3)	
C10	0.5680 (4)	0.69679 (14)	0.93885 (14)	0.0379 (5)	
H10A	0.5624	0.7412	0.8953	0.057*	
H10B	0.6984	0.6895	0.9572	0.057*	
H10C	0.4902	0.7146	0.9863	0.057*	
C11	0.7473 (3)	0.41612 (12)	0.67128 (13)	0.0295 (4)	
H11A	0.7450	0.3999	0.7303	0.044*	
H11B	0.8774	0.4159	0.6511	0.044*	
H11C	0.6729	0.3746	0.6390	0.044*	
C12	0.6677 (3)	0.65206 (11)	0.48211 (11)	0.0214 (3)	
C13	0.6622 (2)	0.74835 (11)	0.48349 (10)	0.0201 (3)	
C14	0.6640 (2)	0.79339 (11)	0.41196 (11)	0.0215 (3)	
H14	0.6658	0.8546	0.4177	0.026*	
C15	0.6619 (3)	0.79287 (11)	0.56742 (11)	0.0224 (3)	
H15A	0.5441	0.7800	0.5966	0.034*	
H15B	0.6732	0.8555	0.5594	0.034*	
H15C	0.7682	0.7720	0.6007	0.034*	
C16	0.6635 (3)	0.75991 (11)	0.32443 (11)	0.0245 (4)	
H16A	0.6156	0.8048	0.2868	0.037*	
H16B	0.5826	0.7087	0.3210	0.037*	
H16C	0.7918	0.7443	0.3081	0.037*	
C17	0.2362 (3)	0.38217 (12)	0.70195 (12)	0.0251 (4)	
H17A	0.165 (3)	0.3387 (14)	0.6752 (13)	0.018 (5)*	
H17B	0.218 (3)	0.3870 (14)	0.7618 (15)	0.028 (6)*	

### Atomic displacement parameters (Å^2^)

**Table:**

	*U*^11^	*U*^22^	*U*^33^	*U*^12^	*U*^13^	*U*^23^
O1	0.0248 (6)	0.0325 (6)	0.0203 (6)	−0.0019 (5)	0.0003 (5)	−0.0013 (5)
O2	0.0399 (8)	0.0341 (7)	0.0234 (6)	0.0069 (6)	0.0046 (6)	0.0021 (5)
O3	0.0268 (6)	0.0167 (5)	0.0218 (6)	0.0001 (5)	0.0052 (5)	0.0007 (5)
O4	0.0485 (8)	0.0216 (6)	0.0247 (6)	−0.0013 (6)	0.0106 (6)	−0.0019 (5)
C2	0.0347 (10)	0.0268 (8)	0.0156 (7)	−0.0025 (8)	0.0019 (7)	0.0039 (6)
C3	0.0355 (10)	0.0264 (9)	0.0214 (8)	−0.0034 (8)	−0.0035 (8)	0.0013 (7)
C3A	0.0269 (9)	0.0224 (8)	0.0219 (8)	−0.0020 (7)	−0.0054 (7)	0.0025 (7)
C4	0.0273 (9)	0.0230 (8)	0.0275 (9)	−0.0057 (7)	−0.0051 (7)	0.0015 (7)
C5	0.0221 (8)	0.0304 (9)	0.0286 (9)	−0.0043 (7)	−0.0033 (7)	0.0025 (7)
C5A	0.0206 (8)	0.0205 (8)	0.0251 (8)	0.0016 (6)	−0.0003 (7)	0.0015 (6)
C6	0.0248 (8)	0.0169 (7)	0.0261 (8)	0.0022 (6)	0.0045 (7)	0.0012 (7)
C7	0.0322 (9)	0.0173 (7)	0.0209 (8)	0.0030 (7)	0.0016 (7)	0.0002 (6)
C8	0.0279 (8)	0.0170 (7)	0.0231 (8)	0.0015 (6)	−0.0018 (7)	0.0004 (6)
C9	0.0216 (8)	0.0207 (8)	0.0230 (8)	0.0005 (7)	−0.0013 (7)	−0.0001 (6)
C9A	0.0211 (8)	0.0184 (7)	0.0188 (7)	0.0001 (6)	−0.0010 (6)	0.0019 (6)
C9B	0.0251 (9)	0.0206 (8)	0.0199 (8)	−0.0018 (7)	−0.0019 (6)	0.0027 (6)
O10	0.0308 (7)	0.0197 (6)	0.0258 (6)	0.0008 (5)	−0.0053 (5)	0.0020 (5)
C10	0.0463 (12)	0.0327 (10)	0.0348 (11)	−0.0077 (9)	0.0000 (10)	−0.0097 (9)
C11	0.0271 (9)	0.0261 (9)	0.0355 (10)	0.0064 (7)	−0.0023 (8)	0.0062 (8)
C12	0.0230 (8)	0.0204 (8)	0.0208 (8)	−0.0017 (7)	0.0022 (7)	−0.0003 (6)
C13	0.0183 (7)	0.0195 (7)	0.0225 (8)	−0.0017 (6)	0.0016 (7)	−0.0004 (6)
C14	0.0196 (8)	0.0200 (7)	0.0248 (8)	−0.0011 (6)	0.0004 (7)	−0.0002 (6)
C15	0.0239 (8)	0.0203 (7)	0.0230 (8)	−0.0013 (7)	0.0010 (7)	−0.0016 (6)
C16	0.0261 (9)	0.0261 (8)	0.0212 (8)	0.0014 (7)	0.0011 (7)	0.0007 (7)
C17	0.0234 (8)	0.0258 (8)	0.0262 (9)	−0.0036 (7)	−0.0017 (7)	−0.0006 (7)

### Geometric parameters (Å, º)

**Table:**

O1—C2	1.349 (2)	C8—O10	1.452 (2)
O1—C9B	1.466 (2)	C8—C9	1.481 (2)
O2—C2	1.206 (2)	C8—H8	1.0000
O3—C12	1.349 (2)	C9—C17	1.341 (3)
O3—C6	1.4510 (19)	C9—C9A	1.514 (2)
O4—C12	1.211 (2)	C9A—C9B	1.527 (2)
C2—C3	1.508 (3)	C9A—H9A	1.0000
C3—C10	1.522 (3)	C9B—H9B	1.0000
C3—C3A	1.533 (3)	C10—H10A	0.9800
C3—H3	1.0000	C10—H10B	0.9800
C3A—C4	1.525 (2)	C10—H10C	0.9800
C3A—C9B	1.535 (2)	C11—H11A	0.9800
C3A—H3A	1.0000	C11—H11B	0.9800
C4—C5	1.526 (3)	C11—H11C	0.9800
C4—H4A	0.9900	C12—C13	1.489 (2)
C4—H4B	0.9900	C13—C14	1.342 (2)
C5—C5A	1.556 (2)	C13—C15	1.512 (2)
C5—H5A	0.9900	C14—C16	1.496 (2)
C5—H5B	0.9900	C14—H14	0.9500
C5A—C6	1.537 (2)	C15—H15A	0.9800
C5A—C11	1.539 (2)	C15—H15B	0.9800
C5A—C9A	1.557 (2)	C15—H15C	0.9800
C6—C7	1.521 (3)	C16—H16A	0.9800
C6—H6	1.0000	C16—H16B	0.9800
C7—O10	1.439 (2)	C16—H16C	0.9800
C7—C8	1.477 (3)	C17—H17A	0.94 (2)
C7—H7	1.0000	C17—H17B	0.97 (2)
			
C2—O1—C9B	110.55 (14)	C17—C9—C8	118.92 (16)
C12—O3—C6	116.01 (13)	C17—C9—C9A	126.23 (16)
O2—C2—O1	121.42 (18)	C8—C9—C9A	114.83 (15)
O2—C2—C3	128.94 (17)	C9—C9A—C9B	113.11 (14)
O1—C2—C3	109.64 (16)	C9—C9A—C5A	110.09 (14)
C2—C3—C10	113.86 (17)	C9B—C9A—C5A	113.57 (14)
C2—C3—C3A	103.43 (14)	C9—C9A—H9A	106.5
C10—C3—C3A	117.94 (17)	C9B—C9A—H9A	106.5
C2—C3—H3	107.0	C5A—C9A—H9A	106.5
C10—C3—H3	107.0	O1—C9B—C9A	105.92 (13)
C3A—C3—H3	107.0	O1—C9B—C3A	106.01 (13)
C4—C3A—C3	116.74 (15)	C9A—C9B—C3A	115.22 (14)
C4—C3A—C9B	110.96 (14)	O1—C9B—H9B	109.8
C3—C3A—C9B	101.65 (15)	C9A—C9B—H9B	109.8
C4—C3A—H3A	109.0	C3A—C9B—H9B	109.8
C3—C3A—H3A	109.0	C7—O10—C8	61.44 (11)
C9B—C3A—H3A	109.0	C3—C10—H10A	109.5
C3A—C4—C5	107.78 (15)	C3—C10—H10B	109.5
C3A—C4—H4A	110.2	H10A—C10—H10B	109.5
C5—C4—H4A	110.2	C3—C10—H10C	109.5
C3A—C4—H4B	110.2	H10A—C10—H10C	109.5
C5—C4—H4B	110.2	H10B—C10—H10C	109.5
H4A—C4—H4B	108.5	C5A—C11—H11A	109.5
C4—C5—C5A	114.37 (16)	C5A—C11—H11B	109.5
C4—C5—H5A	108.7	H11A—C11—H11B	109.5
C5A—C5—H5A	108.7	C5A—C11—H11C	109.5
C4—C5—H5B	108.7	H11A—C11—H11C	109.5
C5A—C5—H5B	108.7	H11B—C11—H11C	109.5
H5A—C5—H5B	107.6	O4—C12—O3	122.45 (16)
C6—C5A—C11	107.32 (15)	O4—C12—C13	125.87 (16)
C6—C5A—C5	109.79 (14)	O3—C12—C13	111.64 (14)
C11—C5A—C5	109.24 (15)	C14—C13—C12	120.38 (15)
C6—C5A—C9A	108.03 (14)	C14—C13—C15	121.65 (15)
C11—C5A—C9A	110.64 (14)	C12—C13—C15	117.93 (15)
C5—C5A—C9A	111.71 (14)	C13—C14—C16	128.49 (15)
O3—C6—C7	110.69 (14)	C13—C14—H14	115.8
O3—C6—C5A	107.57 (13)	C16—C14—H14	115.8
C7—C6—C5A	114.02 (14)	C13—C15—H15A	109.5
O3—C6—H6	108.1	C13—C15—H15B	109.5
C7—C6—H6	108.1	H15A—C15—H15B	109.5
C5A—C6—H6	108.1	C13—C15—H15C	109.5
O10—C7—C8	59.73 (11)	H15A—C15—H15C	109.5
O10—C7—C6	116.08 (13)	H15B—C15—H15C	109.5
C8—C7—C6	120.00 (15)	C14—C16—H16A	109.5
O10—C7—H7	116.3	C14—C16—H16B	109.5
C8—C7—H7	116.3	H16A—C16—H16B	109.5
C6—C7—H7	116.3	C14—C16—H16C	109.5
O10—C8—C7	58.83 (11)	H16A—C16—H16C	109.5
O10—C8—C9	115.17 (14)	H16B—C16—H16C	109.5
C7—C8—C9	120.57 (16)	C9—C17—H17A	122.2 (13)
O10—C8—H8	116.5	C9—C17—H17B	122.1 (14)
C7—C8—H8	116.5	H17A—C17—H17B	115.5 (19)
C9—C8—H8	116.5		
			
C9B—O1—C2—O2	172.91 (16)	O10—C8—C9—C9A	−52.2 (2)
C9B—O1—C2—C3	−8.26 (18)	C7—C8—C9—C9A	15.0 (2)
O2—C2—C3—C10	−28.0 (3)	C17—C9—C9A—C9B	1.8 (3)
O1—C2—C3—C10	153.32 (16)	C8—C9—C9A—C9B	−176.92 (15)
O2—C2—C3—C3A	−157.18 (18)	C17—C9—C9A—C5A	130.09 (19)
O1—C2—C3—C3A	24.10 (18)	C8—C9—C9A—C5A	−48.67 (19)
C2—C3—C3A—C4	92.11 (18)	C6—C5A—C9A—C9	65.44 (17)
C10—C3—C3A—C4	−34.6 (2)	C11—C5A—C9A—C9	−51.76 (19)
C2—C3—C3A—C9B	−28.74 (17)	C5—C5A—C9A—C9	−173.70 (14)
C10—C3—C3A—C9B	−155.42 (17)	C6—C5A—C9A—C9B	−166.57 (14)
C3—C3A—C4—C5	−179.07 (15)	C11—C5A—C9A—C9B	76.22 (19)
C9B—C3A—C4—C5	−63.29 (19)	C5—C5A—C9A—C9B	−45.71 (19)
C3A—C4—C5—C5A	55.2 (2)	C2—O1—C9B—C9A	−133.99 (14)
C4—C5—C5A—C6	118.63 (17)	C2—O1—C9B—C3A	−11.12 (17)
C4—C5—C5A—C11	−123.94 (17)	C9—C9A—C9B—O1	−78.72 (17)
C4—C5—C5A—C9A	−1.2 (2)	C5A—C9A—C9B—O1	154.87 (13)
C12—O3—C6—C7	−75.56 (18)	C9—C9A—C9B—C3A	164.45 (14)
C12—O3—C6—C5A	159.27 (14)	C5A—C9A—C9B—C3A	38.0 (2)
C11—C5A—C6—O3	−166.03 (14)	C4—C3A—C9B—O1	−99.98 (16)
C5—C5A—C6—O3	−47.40 (18)	C3—C3A—C9B—O1	24.83 (16)
C9A—C5A—C6—O3	74.64 (17)	C4—C3A—C9B—C9A	16.8 (2)
C11—C5A—C6—C7	70.81 (18)	C3—C3A—C9B—C9A	141.61 (15)
C5—C5A—C6—C7	−170.56 (14)	C6—C7—O10—C8	−111.03 (17)
C9A—C5A—C6—C7	−48.52 (18)	C9—C8—O10—C7	111.87 (18)
O3—C6—C7—O10	−36.6 (2)	C6—O3—C12—O4	8.5 (3)
C5A—C6—C7—O10	84.87 (18)	C6—O3—C12—C13	−169.25 (15)
O3—C6—C7—C8	−105.14 (17)	O4—C12—C13—C14	16.9 (3)
C5A—C6—C7—C8	16.3 (2)	O3—C12—C13—C14	−165.42 (16)
C6—C7—C8—O10	104.52 (16)	O4—C12—C13—C15	−160.91 (18)
O10—C7—C8—C9	−102.71 (17)	O3—C12—C13—C15	16.8 (2)
C6—C7—C8—C9	1.8 (2)	C12—C13—C14—C16	2.7 (3)
O10—C8—C9—C17	128.89 (18)	C15—C13—C14—C16	−179.63 (18)
C7—C8—C9—C17	−163.85 (17)		

### Hydrogen-bond geometry (Å, º)

**Table:**

*D*—H···*A*	*D*—H	H···*A*	*D*···*A*	*D*—H···*A*
C3*A*—H3*A*···O4^i^	1.00	2.45	3.353 (2)	149
C5—H5*B*···O3	0.99	2.33	2.752 (2)	105
C8—H8···O2^ii^	1.00	2.50	3.176 (2)	124
C9*A*—H9*A*···O3	1.00	2.58	3.010 (2)	106
C14—H14···O10^iii^	0.95	2.57	3.423 (2)	149
C16—H16*B*···O4	0.98	2.45	2.862 (2)	105

Symmetry codes: (i) −*x*+3/2, −*y*+1, *z*+1/2; (ii) −*x*+1/2, −*y*+1, *z*−1/2; (iii) *x*+1/2, −*y*+3/2, −*z*+1.
